# Effect of inorganic phosphate on migration and osteogenic differentiation of bone marrow mesenchymal stem cells

**DOI:** 10.1186/s12861-020-00229-x

**Published:** 2021-01-06

**Authors:** Hengzhang Lin, Yong Zhou, Qun Lei, Dong Lin, Jiang Chen, Chuhuo Wu

**Affiliations:** 1 Department of Stomatology, Fujian Provincial Governmental Hospital, Fuzhou, China; 2 Fujian Key Laboratory of Oral Diseases & Fujian Provincial Engineering Research Center of Oral Biomaterial & Stomatological Key Laboratory of Fujian College and University, School and Hospital of Stomatology, Fujian Medical University, Fuzhou, China; 3 Fujian Medical University, Fuzhou, China

**Keywords:** Phosphate, Mesenchymal stem cells, Bone defect, Bone substitute materials, Implant

## Abstract

**Background:**

Phosphate is the major ingredient of bone tissue, and is also an important component of commercial bone substitute materials, bone scaffolds, and implant surface coatings. With the dissolution of the bone substitute materials and the degradation by cells, local ion concentrations will change and affect bone tissue reconstruction. Bone marrow -derived mesenchymal stem cells (BM-MSCs) are main autologous cells to repair injured bone. When bone injure occurs, BM-MSCs migrate to the damaged area, differentiate into osteoblasts, and secrete bioactive factors to promote bone tissue repaired. This study aimed to investigate the effect of inorganic phosphate (Pi) at a series of concentration on migration and osteogenic differentiation of human bone marrow -derived mesenchymal stem cells(hBM-MSCs).

**Methods:**

The culture of hBM-MSCs in mediums with different concentration of Pi from 2 mM to 10 mM were performed. HBM-MSCs migration were examined with transwell assays. HBM-MSCs proliferation were evaluated by cell counting kit-8 colorimetric method. Osteogenic genes expression were analyzed by real-time reverse transcriptase polymerase chain reaction. Mineralized nodules formation were demonstrated by Alizarin red staining.

**Result:**

4–10 mM Pi could effectively promote the migration of hBM-MSCs at 12 h and 18 h. There was no significant difference in the migration number of hBM-MSCs in Pi culture mediums at a concentration of 6, 8, and10mM. 2–10 mM Pi could promote the proliferation of hBM-MSCs to varying degrees in the observation period, while 4–10 mM Pi could promote the osteogenic differentiation and mineralization of hBM-MSCs.

**Conclusion:**

The findings in our study showed 4-10 mM Pi could promote the migration, osteogenic differentiation, and mineralization of hBM-MSCs.

**Supplementary Information:**

The online version contains supplementary material available at 10.1186/s12861-020-00229-x.

## Background

Trauma, tumor resection, disuse atrophy, congenital defects, systemic diseases often result in jaw defect. Due to limited bone mass and possible adverse postoperative reactions, autogenous bone transplantation has been unable to meet the need of bone defects. The application of commercial bone substitute materials have largely solved the problem of insufficient bone mass, on the other hand, with the dissolution of the bone substitute materials and the degradation by cells, local ions concentration will change and affect bone tissue reconstruction [1–3]. Understanding the effect of the components of bone substitute materials on the biological behavior of cells are critical to the construction of materials. At the same time, compared with growth factors and genes regulation, natural or chemically synthesized small molecules can induce the directional differentiation of stem cells and accelerate the reconstruction and repair of the bone more economically and effectively [4].

Phosphate is the major ingredient of bone tissue, and is also an important component of commercial bone substitute materials, bone scaffolds, and implant surface coatings [5–7]. Inorganic phosphate (Pi) in organisms exists in the form of H_2_PO_4_^−^ and HPO_4_^2−^.Extracellular Pi can stimulate signal transduction and regulate the functions of osteoblasts and stem cells. Human bone marrow -derived mesenchymal stem cells (hBM-MSCs) are main autologous cells to repair injured bone. When bone injure occurs, hBM-MSCs migrate to the damaged area, differentiate into osteoblasts, and secrete bioactive factors to promote bone tissue repair [8]. To explore the effects of Pi on the migration, proliferation and osteogenic differentiation of hBM-MSCs, it has certain guide significance for bone tissue regeneration. On one hand, the Pi released after material degradation in vivo can be controlled to facilitate the reconstruction of surrounding bone tissue. On the other hand, with the discovery of drug delivery systems in vivo, Pi can be loaded or transported to the bone defect via slow released macromolecule carrier, which concentration can be adjusted in tissue microenvironment to accelerate bone tissue reconstruction [9–11].

Although the functions of phosphorus-containing biomaterials have been studied to a certain extent, the research objects are mainly phosphorus-containing complexes, which have complex composition and diverse structure. It is difficult to distinguish the effect on cells derived from the morphology or the specific substance. Most of cells used in previous studies are animal-derived cells, and there are also certain differences in the response to materials between animal-derived cells and human-derived cells. Therefore, the effect of Pi on the biological behavior of hBM-MSCs was explored in this study.

## Methods

### Preparation of pi containing medium

To prepare 5 different concentrations of Pi, Sodium dihydrogen phosphate dihydrate (NaHPO_4_ • 2H_2_O; Shanghai Hengyuan Biotechnology Co., Ltd., China) and disodium hydrogen phosphate dihydrate(NaH_2_PO_4_ • 2H_2_O; Shanghai Hengyuan Biotechnology Co., Ltd., China) were dissolved in HEPES buffer solution at a ratio of 4: 1 [12] to make Pi stock solution concentration of 200, 400, 600, 800, 1000 mM. Pi culture mediums concentration of 2, 4, 6, 8, 10 mM (2mMPi, 4mMPi, 6mMPi, 8mMPi, 10mMPi) were prepared by serially diluting stock solutions in mesenchymal stem cell growth medium (Guangzhou Saiye Biotechnology Co., Ltd., China). hBM-MSCs(HUXMA-01001, Guangzhou Saiye Biotechnology Co., Ltd., China) incubated in mesenchymal stem cell growth medium (GM) were applied as a control.

### HBM-MSCs migration assay

HBM-MSCs migration were evaluated using transwell assay in 24-well plates (Corning, USA). The lower chambers were filled with Pi culture mediums at concentration of 2, 4, 6, 8, or 10 mM as a chemoattractant, while they were filled with mesenchymal stem cell growth medium in control group. HBM-MSCs (Guangzhou Saiye Biotechnology Co., Ltd., China) were suspended with mesenchymal stem cell growth medium at a concentration of 1 × 10^4^/ ml cells and added to the upper chambers. The transwell plates were incubated at 37 °C, 5% CO_2_ for 6, 12, or 18 h, respectively. Then the residue cells in upper chambers were removed, cells in transwell membranes close to the lower chambers were fixed with 10% formalin, stained with DAPI and observed under fluorescent microscope (Zeiss, Germany). Cells in ten independent, randomly-chosen fields of view were counted as the average number of migrated cells. All experiments were performed in triplicate.

### HBM-MSCs proliferation

The hBM-MSCs seeded onto 96-well plates at a density of 1 × 10^4^ / cm^2^ cells were cultured with mesenchymal stem cell growth medium at 37 °C, 5% CO_2_. After 24 h, the media was replaced with Pi culture mediums at a concentration of 2, 4, 6, 8, or 10 mM, respectively. Mesenchymal stem cell growth medium was applied as a control. To evaluated cell proliferation, hBM-MSCs were incubated in different mediums for 1, 4, 7, or 10d, respectively, and then examined by cell counting kit-8 colorimetric method (CCK8; Beijing Taize Ruida Technology Co., Ltd., China) according to the manufacturer’s instructions. Five repeats were performed in the experiments.

### HBM-MSCs osteogenic differentiation

#### Osteocalcin (OC), alkaline phosphatase (ALP), collagen type I (COLI) gene expression

The hBM-MSCs seeded onto 6-well plates at a density of 1 × 10^4^ / cm^2^ cells were cultured with mesenchymal stem cell growth medium at 37 °C, 5% CO_2_ for 24 h, and then the media was replaced with Pi culture mediums at a concentration of 2, 4, 6, 8, or 10 mM, respectively. Mesenchymal stem cell growth medium was applied as a control. After hBM-MSCs were incubated in different mediums for 4, 7, or 10d, respectively, the analysis about genes expression of OC, ALP, and COL I were performed by real-time reverse transcriptase polymerase chain reaction (RT-PCR). RNA was extracted by RNeasy Mini Kit (Qiagen, Germany). cDNA was synthesized by Takara RR047A reverse transcription kit (Takara, Japan). The quality and quantity of RNA and cDNA were detected by Nano Drop2000 (Thermo, USA). SYBR® Green Real-Time PCR Master Mix (Toobo, Japan) was used to quantified transcript levels of OC, ALP, and COL I genes. Genes expression were analyzed according to the relative quantification 2^−ΔΔCt^ method. All experiments were conducted in triplicate. Glyceraldehyde-3-phosphate dehydrogenase (*GAPDH*) was used as an internal control to normalize transcript levels of OC, ALP, and COL I genes. The primer sequences designed for GAPDH, OC, ALP, and COL I genes are as follows: GAPDH sense strand: 5′-GGTTGTCTCCTGCGACTTCA-3 ‘, antisense strand: 5’-TGGTCCAGGGTTTCTTACTCC-3′; OC sense strand: 5′-CAGGCGCTACCTGTATCAATG-3 ‘, antisense strand: 5’-GATGTGGTCAGCCAACTCGT-3 ‘. ALP sense strand: 5’-AACATCAGGGACATTGACGTG-3 ‘, antisense strand: 5’-GTATCTCGGTTTGAAGCTCT 3′; COL I sense strand: 5′-AGACATCCCACCAATCACCTG-3 ‘, antisense strand: 5’-CGTCATCGCACAACACCTT-3′.

### Mineralized nodules staining

The hBM-MSCs seeded onto 24-well plates at a density of 1 × 10^4^ / cm^2^ cells were cultured with mesenchymal stem cell growth medium at 37 °C, 5% CO_2_. After 24 h, the media was replaced with Pi culture mediums at a concentration of 2, 4, 6, 8, or 10 mM, respectively. Mesenchymal stem cell growth medium was applied as a control, while Pi culture mediums at a concentration of 2, 4, 6, 8, or 10 mM without cells were used as blank controls. The cells were further cultured in different culture mediums for 10 d, and then fixed in 95% ethanol for 10 min and stained with 0.5% Alizarin Red-Tris-HCl (Sigma, USA) at room temperature for 30 min. The plates were washed with distilled water and then images were taken under optical microscopy (Zeiss, Germany).

### Statistical analysis

Statistical analysis was performed using SPSS 20.0. One-way ANOVA was used to analyze the differences among the groups. The experimental data were presented as mean ± standard deviation, and *P* < 0.05 was considered as statistically significant.

## Results

### HBM-MSCs migration

**Fig. 1:**
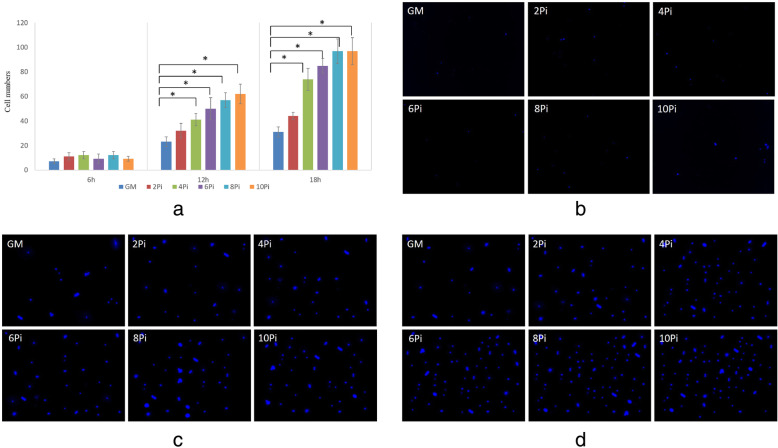
Transwell migration assay was conducted on hBM-MSCs treated with 2-10mMPi mediums or GM. **a** The cell numbers for migration in 2-10mMPi mediums and GM over time (6 h, 12 h, 18 h). ‘*’ showed statistical significance with *p* value < 0.05 when compared to GM group. **b** The observation on the migration of hBM-MSCs at 6 h under fluorescence inverted microscope (× 400) **c** The observation on the migration of hBM-MSCs at 12 h under fluorescence inverted microscope (× 400) **d** The observation on the migration of hBM-MSCs at 18 h under fluorescence inverted microscope (× 400)

To elucidate the potential role of Pi on hBM-MSCs migration, transwell migration assay was performed. As shown in Fig. 1, no significant difference in the migration of hBM-MSCs was detected after migration was allowed to proceed for 6 h in each experimental group, and yet, 4–10 mM Pi were found to promote the migration of hBM-MSCs (*P* < 0.05) in the next 12 h to 18 h. The cell numbers for migration in 6-10mMPi mediums had no significant difference after cultivated for 12 h and 18 h.

### HBM-MSCs proliferation

**Fig. 2:**
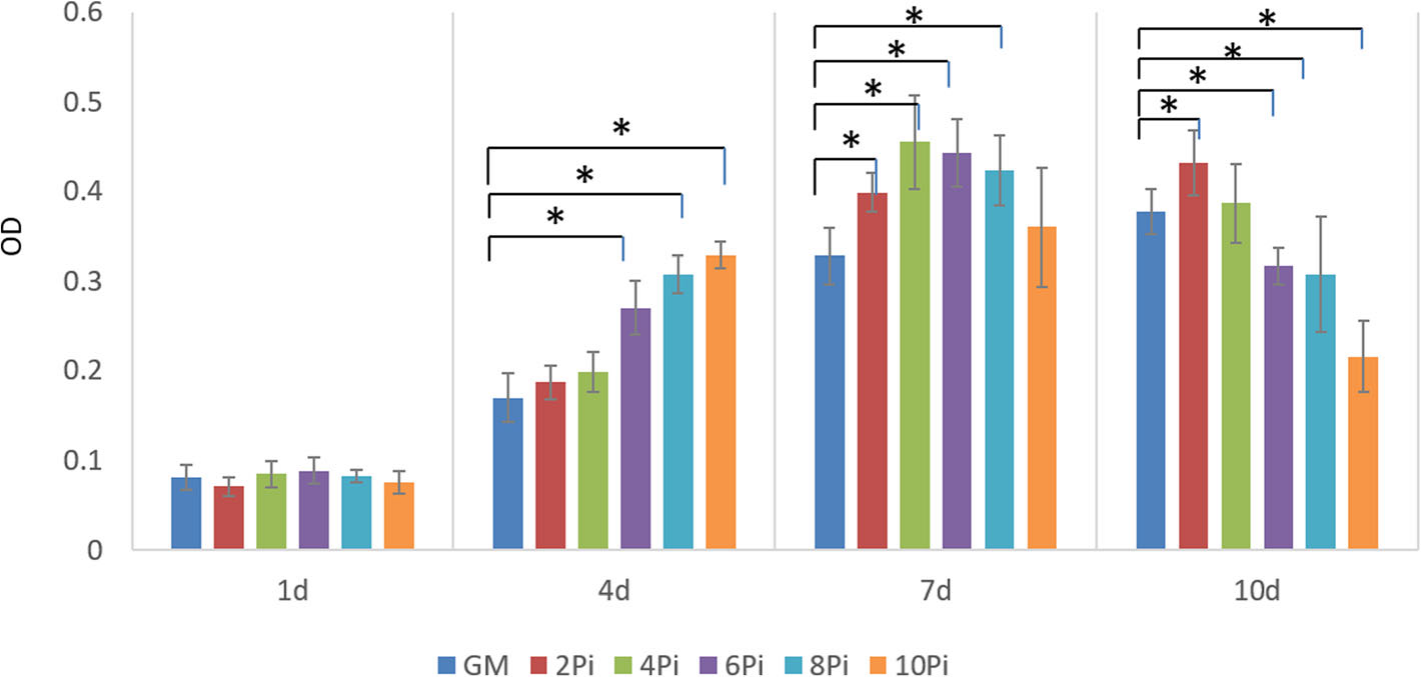
CCK-8 cell proliferation assay was performed on hBM-MSCs treated with 2-10mMPi mediums or GM on days 1,4,7,10. ‘*’ showed statistical significance with *p* value < 0.05 when compared to GM group

To understand the effect of Pi on hBM-MSCs proliferation, CCK-8 assay was conducted on cells treated with GM or Pi culture medium. The results showed there were no significant difference in the cells proliferation between the cells cultured in GM and Pi culture mediums on day 1. The number of hBM-MSCs in the 6-10mMPi mediums were significantly higher than those in the GM after 4 days. The number of hBM-MSCs in the 2-8mMPi mediums were higher than those in the GM after 7 days. On day 10, the number of hBM-MSCs in the 2mMPi medium were higher than those in the GM, while the number of hBM-MSCs in the 6-10mMPi mediums were lower than those in the GM (Fig. 2).

### HBM-MSCs osteogenic differentiation

**Fig. 3:**
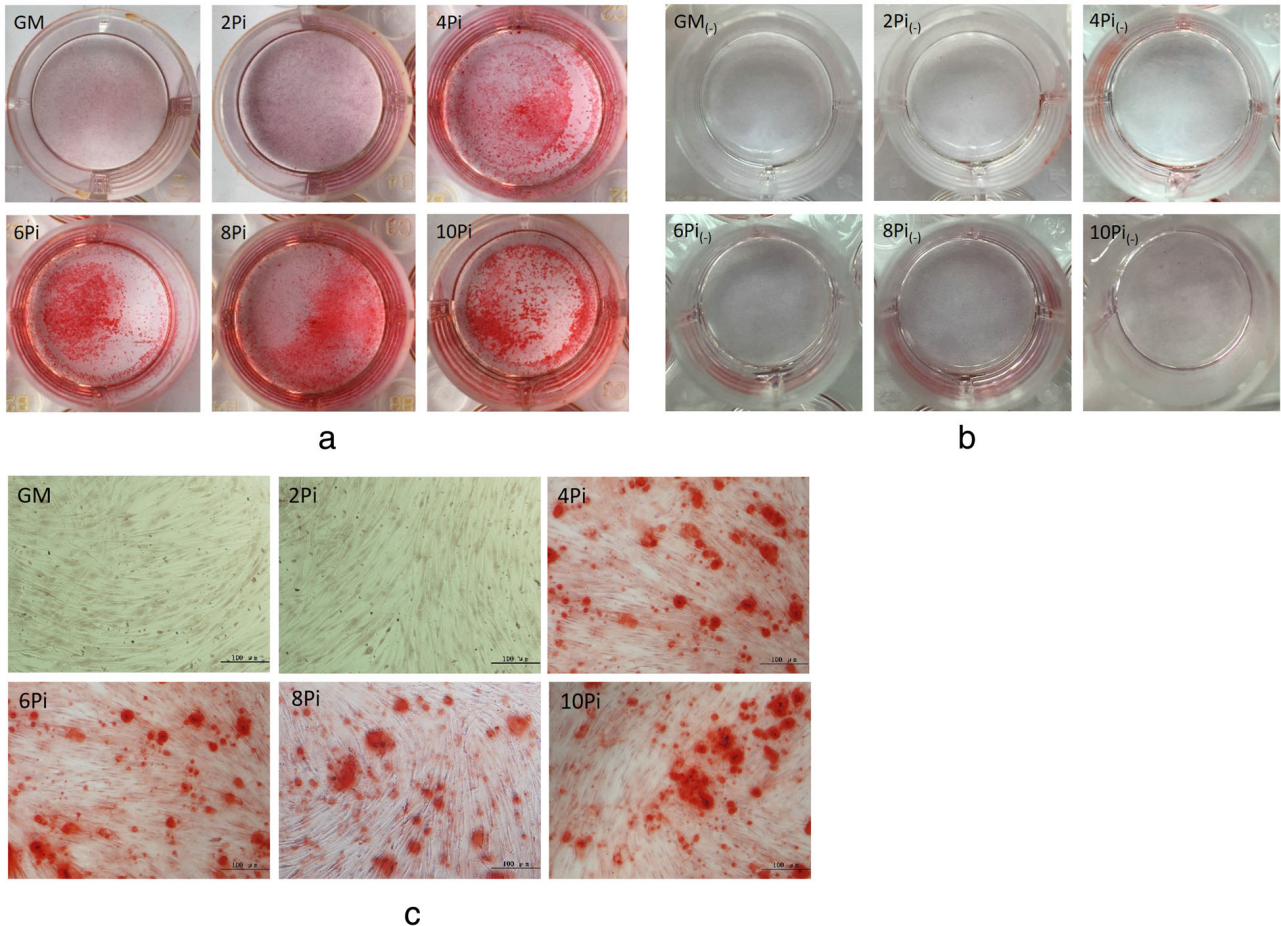
Alizarin red staining of mineralized nodules. **a** Alizarin Red S coloration showed the mineralized nodules deposit in 4-10mMPi mediums under naked eye observation. **b** Alizarin Red S coloration demonstrated no mineralized nodules deposit in 2-10mMPi mediums without cells (2Pi_(−),_ 4Pi_(−)_,6Pi_(−)_,8Pi_(−)_,10Pi_(−)_) **c** Alizarin Red S coloration showed the mineralized nodules deposit in 4-10mMPi mediums under microscope(× 100)

**Fig. 4:**
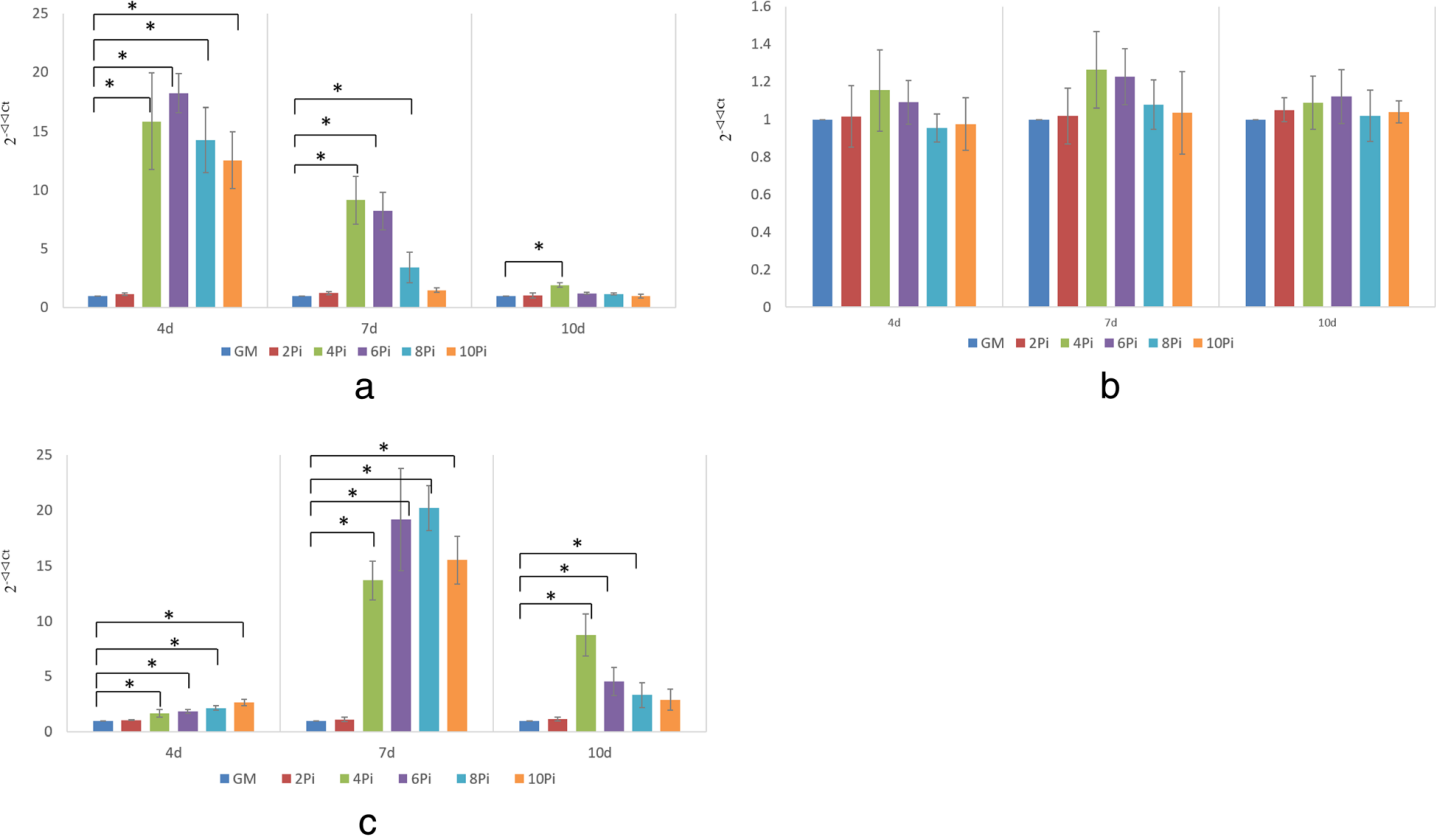
RT-PCR analysis about genes expression of COL I, ALP, and OC in 2-10mMPi mediums and GM over time (4d, 7d, 10d). ‘*’ showed statistical significance with *p* value < 0.05 when compared to GM group. **a** COL I gene expression; **b** ALP gene expression; **c** OC gene expression

Osteogenic differentiation of hBM-MSCs were evaluated by the mineralization nodules staining with Alizarin Red S and genes expression of COLI, ALP and OC. Figure 3a and Fig. 3c demonstrated that while the cells in GM and 2mMPi mediums did not create the deposit after 10 days of incubation, the cells in 4-10mMPi mediums formed mineralized nodules deposit. Figure 3b showed deposit formation was not visible in 2-10mMPi mediums without cells seeded. These results were proved by the RT-PCR findings (Fig. 4). There were no significant difference in genes expression of COLIand OC between the cells in GM and cells in 2mMPi medium, while the cells in 4-10mMPi mediums highly expressed the COLIand OC genes in varying degree at the time of day 4, day7, or day 10. We did not observe the significant difference in the gene expression of ALP between the cells in GM and cells in Pi culture mediums.

## Discussion

HBM-MSCs are main autologous cells to repair bone defect, and also widely used as seed cells in bone tissue engineering and regenerative medicine. Pi is an important component in the formation of hydroxyapatite. Approximately 85% of the Pi is stored in the extracellular matrix of bone and teeth in the form of hydroxyapatite, 14% of the Pi exists in the cells in the form of polymer, covalent binding, free state, etc., and only 1% of the Pi exists in the extracellular fluid to maintain cell metabolism, among which, the concentration of free Pi in intracellular fluid is approximately equal to that in the extracellular fluid [13, 14]. The Pi concentration in children’s serum is approximately 4.88 ± 0.66 mg / dl (1.58 ± 0.21 mM), while it is in adult serum is approximately 3.63 ± 0.51 mg / dl (1.17 ± 0.16 mM) [15]. Pi is also an important component of commercial bone replacement materials, bone scaffold materials, and implant surface coatings. Locally sustained Pi release can advance collagen mineralized, accelerate bone healing, and enhance osseointegration of implants. There is certain guiding significance for the improvement and optimization of bone regeneration materials and implant surface coatings to explore the Pi concentration range to promote migration, proliferation and osteogenic differentiation of hBM-MSCs.

In migration experiments, it was observed that 4–10 mM Pi could effectively promote the migration of hBM-MSCs at 12 h and 18 h (*P* < 0.05). Among them, there were no significant difference in the migration number of hBM-MSCs in Pi culture mediums at a concentration of 6, 8, and10mM, suggesting that the ability of 6-10 mM Pi to promote the migration of hBM-MSCs were similar. At 24 h, there were no significant difference in the proliferation of hBM-MSCs in different experimental groups, indicating that the increase in the number of hBM-MSCs in the early stage were due to the effect of Pi in promoting cells migration, and had nothing to do with cells proliferation. The number of hBM-MSCs in the 6–10 mM Pi mediums were higher than those in the GM on day 4; the number of hBM-MSCs in the 2–8 mM Pi mediums were higher than those in the GM on day 7; only the number of hBM-MSCs in 2 mM Pi medium were higher on day 10, while the number of hBM-MSCs in the 6–10 mM Pi mediums were lower than those in the GM. Staining experiments of mineralized nodules showed that there were red stained mineralized nodules deposit in the 4-10mMPi mediums after 10d. In the blank control groups, no red-stained mineralized nodules generated, indicating that the deposited mineralized nodules originated from hBM-MSCs after osteogenic differentiation, rather than from Pi deposition in mediums. The lower number of hBM-MSCs found in 6–10 mM Pi mediums may be caused by the fact that some cells were in the mineralization stage. No red-stained mineralized nodules formed in the GM and 2mMPi mediums, at the same time, there were no significant difference in the expression of COLIand OC genes in these groups, indicating that 2mMPi might not promote osteogenic differentiation of hBM-MSCs. During the observation period, the expressions of COLIand OC genes increased to varying degrees in the 4-10mMPi mediums, but the expression of ALP gene did not change significantly. Bone mineralization begins with matrix vesicles (MVs), which exist in the plasma membrane of osteoblasts and chondrocytes. MVs contain highly active tissue non-specific ALP, which can hydrolyze phosphate esters such as pyrophosphate to generate endogenous Pi. The extracellular Pi can be transported to the cell through co-transporters, which is absorbed by MVs and eventually exists in the form of hydroxyapatite. Presumably, the increase of exogenous Pi may inhibit the generation of endogenous Pi which can be produced by hydrolysis of phosphate esters through ALP [16, 17].

Sai et al. found that, due to Pi released from octacalcium phosphate (OCP) during the hydrolysis process, OCP could promote the differentiation of mouse-derived osteoblasts better than hydroxyapatite and tricalcium phosphate. The elevated Pi concentration in medium promoted the differentiation of osteoblasts. Further research showed that, in the absence of OCP, a medium with Pi concentration up to 1.5 mM could also promote osteoblast differentiation, while inhibiting intracellular transport of Pi, the effect of inducing osteoblast differentiation would be offset [18]. Ali Akbari Ghavimi et al. observed 1–8 mM Pi had an active effect on the proliferation and differentiation of mouse-derived bone marrow mesenchymal stem cells [19]. Nevertheless, in our experiment, 2 mM Pi had no obvious osteoinduction on hBM-MSCs, which may be related to the different ethnic origin of cells.

Biomineralization is a complex process. At present, there are mainly three mechanisms: (1) MVs exist in the plasma membrane of mineralized cells, and they can take up phosphate ions and calcium ions to provide a microenvironment for the formation of apatite, which is then released and deposited in the extracellular matrix. (2) Phosphate ions and calcium ions can enter the mineralized cells through the channels on the cells membrane, and then be enriched in the intracellular vesicles to form amorphous apatite particles, which can be transported and deposited on the collagen fiber after vesicle budding, and finally form high crystallite apatite. (3) The collagen gap zones of the extracellular matrix are directly nucleated by non-collagen-mediated minerals. Non-collagen proteins mainly include dentin matrix protein 1, dentin sialophosphoprotein, etc. Currently, most studies support the first mechanism [20, 21].

During biomineralization, large amounts of Pi must be transported from the circulatory system into osteoid. This process requires multiple signaling pathways to participate, and Pi transport requires the synergistic action of co-transporters. There are various sodium–phosphate co-transporters on the surface of mineralized cells, such as NaPi-IIa (SLC34A1), NaPi-IIb (SLC34A2), Pit-1 (SLC20A1), Pit-2 (SLC20A2) [21–23]. During osteogenic differentiation of cells, Pi can be transported from extracellular to intracellular by these transporters and initiate multiple signaling pathways. Hydroxyapatite formation and biomineralization are closely related to these transporters and signal transduction [24–27].

In order to apply Pi more effectively, the transport and sustained-release of Pi are the key problems to be solved in its clinical application. Multiple delivery systems are under study [28–31], and those findings bring hope to us. Polyphosphate, a linear polymer of orthophosphates which can be polymerized to length of hundreds of phosphates, can be used as an effective source of Pi [32]. Guanosine 5′-diphosphate (GDP) cross-linked chitosan sponge is an injectable and degradable polymer material which can be used as a carrier of drug delivery system. Pyrophosphatase can be sealed into GDP cross-linked chitosan sponge to form a complex. When the complex is placed into the medium, GDP will exudate from the sponge and be decomposed into guanosine and pyrophosphate by the ALP generated through osteoblast differentiation. Pyrophosphate can be cracked into Pi under the action of released pyrophosphatase, so as to achieve the release of Pi [11]. Oligo [(polyethylene glycol) fumarate] (OPF) hydrogels is a polymer based on polyethylene glycol, which has good biocompatibility, injectability and biodegradability, and can be used as drug carrier. OPF itself has no effect on promoting osteoblast adhesion, proliferation and osteogenic differentiation. However, the OPF hydrogel modified by Pi was found to promote the attachment, proliferation and differentiation of osteoblasts and accelerate bone healing [9, 10]. So, the next focus of our research is to select appropriate sustained-release carrier that can load or transport Pi to the bone defect of the jaw and release the effective concentration.

## Conclusion

This study investigated the effect of 2 to 10 mM Pi on migration, proliferation, and osteogenic differentiation of hBM-MSCs. It was found that 2 to 10 mM Pi could promote the proliferation of hBM-MSCs, while 4 to 10 mM Pi could promote migration, osteogenic differentiation and mineralization of hBM-MSCs.

## Supplementary Information

**Additional file 1.**

## Data Availability

All data generated or analysed during this study are included in this published article [and its supplementary information files]. The datasets used and/or analysed during the current study available from the corresponding author on reasonable request.

## Abbreviations

Pi: Inorganic phosphate
hBM-MSCs: Human bone marrow -derived mesenchymal stem cells
NaHPO_4_ • 2H_2_O: Sodium dihydrogen phosphate dihydrate
NaH_2_PO_4_ • 2H_2_O: Disodium hydrogen phosphate dihydrate
GM: Growth medium
CCK8: Counting kit-8 colorimetric method
OC: Osteocalcin
ALP: Alkaline phosphatase
COL I: Collagen type I
RT-PCR: reverse transcriptase polymerase chain reaction
GAPDH: Glyceraldehyde-3-phosphate dehydrogenase
MVs: Matrix vesicles
OCP: Octacalcium phosphate
GDP: Guanosine 5′-diphosphate
OPF: Oligo [(polyethylene glycol) fumarate]
