# Supplementary material for: Effect of inorganic phosphate on migration and osteogenic differentiation of bone marrow mesenchymal stem cells
Source: BMC Dev Biol. 2021 Jan 6;21:1. doi: 10.1186/s12861-020-00229-x (PMC7788862; doi:10.1186/s12861-020-00229-x)
Supplement: Supplementary file 1 — Additional file 1. [file 12861_2020_229_MOESM1_ESM.doc]

**Results**

1. **HBM-MSCs migration**

|  | GM | 2P | 4P | 6P | 8P | 10P |
| --- | --- | --- | --- | --- | --- | --- |
| 6h | 7±2 | 11±3 | 12±3 | 9±4 | 12±3 | 9±2 |
| 12h | 23±4 | 32±6 | 41±5* | 50±9* | 57±6* | 62±8* |
| 18h | 31±4 | 44±3 | 74±9* | 85±6* | 97±10* | 97±11* |

1. **HBM-MSCs proliferation**

|  | GM | 2P | 4P | 6P | 8P | 10P |
| --- | --- | --- | --- | --- | --- | --- |
| 1d | 0.082±0.014 | 0.071±0.010 | 0.085±0.015 | 0.089±0.015 | 0.083±0.007 | 0.076±0.013 |
| 4d | 0.170±0.027 | 0.187±0.019 | 0.199±0.022 | 0.270±0.030* | 0.308±0.021* | 0.329±0.015* |
| 7d | 0.328±0.031 | 0.399±0.021* | 0.455±0.052* | 0.443±0.038* | 0.423±0.039* | 0.360±0.066 |
| 10d | 0.377±0.025 | 0.432±0.036* | 0.387±0.044 | 0.317±0.020* | 0.308±0.064* | 0.216±0.040* |

1. **HBM-MSCs osteogenic differentiation**

|  | GM | 2P | 4P | 6P | 8P | 10P |
| --- | --- | --- | --- | --- | --- | --- |
| 4d | 1 | 1.123±0.108 | 15.852±4.107* | 18.222±1.657* | 14.257±2.759* | 12.533±2.404* |
| 7d | 1 | 1.225±0.137 | 9.154±2.036* | 8.214±1.613* | 3.423±1.285* | 1.482±0.180 |
| 10d | 1 | 1.037±0.214 | 1.918±0.176* | 1.214±0.074 | 1.164±0.092 | 0.961±0.171 |

1. gene expression of COLⅠ

|  | GM | 2P | 4P | 6P | 8P | 10P |
| --- | --- | --- | --- | --- | --- | --- |
| 4d | 1 | 1.016±0.163 | 1.153±0.216 | 1.090±0.114 | 0.954±0.076 | 0.975±0.139 |
| 7d | 1 | 1.017±0.146 | 1.264±0.203 | 1.225±0.150 | 1.078±0.129 | 1.035±0.219 |
| 10d | 1 | 1.050±0.063 | 1.088±0.141 | 1.121±0.142 | 1.018±0.136 | 1.039±0.057 |

1. gene expression of ALP

|  | GM | 2P | 4P | 6P | 8P | 10P |
| --- | --- | --- | --- | --- | --- | --- |
| 4d | 1 | 1.064±0.038 | 1.660±0.330* | 1.823±0.165* | 2.133±0.201* | 2.656±0.308* |
| 7d | 1 | 1.119±0.194 | 13.671±1.752* | 19.165±4.623* | 20.205±2.027* | 15.509±2.137* |
| 10d | 1 | 1.151±0.168 | 8.737±1.907* | 4.558±1.268* | 3.331±1.129* | 2.895±0.939 |

1. gene expression of OC
